# Recombinant *Salmonella* expressing SspH2-EscI fusion protein limits its colonization in mice

**DOI:** 10.1186/s12865-017-0203-2

**Published:** 2017-05-03

**Authors:** Maozhi Hu, Weixin Zhao, Wei Gao, Wenhua Li, Chuang Meng, Qiuxiang Yan, Yuyang Wang, Xiaohui Zhou, Shizhong Geng, Zhiming Pan, Guiyou Cui, Xinan Jiao

**Affiliations:** 1 Jiangsu Co-innovation Center for Prevention and Control of Important Animal Infectious Diseases and Zoonoses, Yangzhou University, Yangzhou, 225009 Jiangsu China; 2 Jiangsu Key Laboratory of Zoonosis, Yangzhou University, Yangzhou, 225009 Jiangsu China; 3 College of Tourism & Cuisine (College of Food Science and Engineering), Yangzhou University, Yangzhou, 225009 Jiangsu China; 4 Department of Pathobiology and Veterinary Science, University of Connecticut, Storrs, 06269-3089 CT USA

**Keywords:** *Salmonella*, Inflammasome, SspH2-EscI, Mice, Colonization

## Abstract

**Background:**

Activation of inflammasome contributes to the clearance of intracellular bacteria. C-terminus of *E. coli* EscI protein can activate NLRC4 (NLR family, CARD domain containing-4) inflammasome in macrophages. The purpose of this study was to determine if activation of NLRC4 inflammasome by EscI can reduce the colonization of *Salmonella* in mice.

**Results:**

A recombinant *S. typhimurium* strain expressing fusion protein of the N-terminal SspH2 (a *Salmonella* type III secretion system 2 effector) and C-terminal EscI was constructed and designated as X4550(pYA3334-SspH2-EscI). In vitro assay showed that X4550(pYA3334-SspH2-EscI) significantly enhanced IL-1β and IL-18 secretion (*P* < 0.05) and pyroptotic cell death of mouse peritoneal macrophages, compared with those infected with control strain, X4550(pYA3334-SspH2). In vivo studies showed that colonization of X4550(pYA3334-SspH2-EscI) in both spleen and liver were significantly lower than that of X4550(pYA3334-SspH2) (*P* < 0.05). The bacterial counts of X4550(pYA3334-SspH2-EscI) in mice decreased, while those of X4550(pYA3334-SspH2) increased over the time after infection. Additionally, X4550(pYA3334-SspH2-EscI) induced a less pathological alteration in spleen and liver than X4550(pYA3334-SspH2).

**Conclusion:**

Fusion protein SspH2-EscI may be translocated into macrophages and activate NLRC4 inflammasome, which limits *Salmonella* colonization in spleen and liver of mice.

## Background

Innate immune system plays a primary role in the rapid elimination of invading microorganisms, which occurs via the recognition of microbial pathogen-associated molecular patterns by the cellular pattern recognition receptors [1, 2]. Intracellular nucleotide binding domain leucine-rich repeat-containing receptor (NLR) can recognize microbial components that are transported to the cytoplasm through the bacterial secretion system, which can then activate inflammasome signalling [3, 4]. During this process, pro-caspase-1 is synthesized by activated macrophages and enriched in the inflammasome, ultimately being cleaved into the activated caspase-1 [5]. Activation of caspase-1 subsequently triggers IL-1β/IL-18 maturation and macrophage pyroptotic death. This pathway is important to defend against the colonization of intracellular bacteria in the intestinal tract and systemic circulation [6–8].

After infection, *Salmonella* can selectively secret cytoplasmic effectors through its type III secretion system (T3SS) [9–11]. These effectors regulate the host cells’ defense mechanisms to ensure the survival of the invading bacteria [12, 13]. Infection of *Salmonella* can result in both decreased breeding potential and increased fatality in host organism. Therefore, a key defensive step to protect against *Salmonella* infection is to reduce bacterial intracellular survival. Currently, there is no effective approach to induce adaptive immunity during the early stages of a *Salmonella* infection, thus it is critical to focus on innate immunity.

During the early stage of infection, *Salmonella* T3SS1 effectors are expressed to mediate bacterial infection. Once *Salmonella* enters host cells, T3SS2 effectors are expressed in order to mediate bacterial intracellular survival [14]. Though many proteins of *Salmonella* can activate intracellular inflammasome response, over the course of evolution, *Salmonella* has developed the ability to escape the inflammasome responses. This can occur through the T3SS1 protein PrgJ that can activate the NLRC4 inflammasome in macrophages, but is only expressed during the early stage of infection. When *Salmonella* successfully survives intracellularly, it no longer express PrgJ [15]. This suggests that if *Salmonella* strain can persistently express and transport PrgJ to the cytoplasm of host cells, it can enhance the activation of inflammasome and thereby inhibit the intracellular survival of bacteria [16]. It has also been reported that the immunization of the *Listeria monocytogenesis* strain that can enhance caspase-1 activation can confer protective immunity against a subsequent wild-type challenge [17]. Thus, it has been hypothesized that the *Salmonella* strain with the ability to enhance caspase-1 activation can strengthen the cell’s defense against *Salmonella* infection [18].

Previous reports have suggested that inflammasome activation mechanism can be used in the design of recombinant vaccines to limit the colonization of intracellular bacteria in vivo [19]. As previously reported, the N-terminus signal peptide of the *Salmonella* effector SspH2 can be recognized by T3SS2 and transported into the cytoplasm [20, 21]. The C-terminus of *E. coli* EscI protein can activate the NLRC4 (NLR family, CARD domain containing-4) inflammasome in macrophages [15]. In the present study, a recombinant *Salmonella* fusion expressing the N-terminus of *Salmonella* SspH2 and the C-terminus of *E. coli* EscI was constructed. The recombinant strain was tested for its ability to activate inflammasome and colonize in vivo in mouse.

## Methods

### Animals, plasmids and bacteria

Six-week-old female C57BL/6 mice were obtained from the Comparative Medicine Center of Yangzhou University (Yangzhou, China). This study was carried out in accordance with the regulations established by the Chinese Ministry of Science and Technology. The animal experiment protocol was approved by the Committee on the Ethics of Animal Experiments of Yangzhou University (Permit Number: 2007–0005). All surgery was performed under sodium pentobarbital anesthesia, and all efforts were made to minimize suffering.

Plasmids pMD20 T (Amp^+^) and pYA3334 (asd^+^), *E. coli* DH5α (RˉMˉ, Ampˉ) and X6212 (asdˉ, NA^+^, RˉM^+^), attenuated *S.* Typhimurium strains X3730 (GalEˉ, Hsdˉ, Asdˉ, NA^+^, RˉM^+^) and X4550 (△crp-1, △cya-1, asdˉ, NA^+^, RˉM^+^) were used in this study as previously described [22, 23]. *S.* Enteritidis C50041 and *E. coli* O:157 were used for the amplification of *sspH2* and *escI* genes, respectively. Bacterial strains were grown in Luria broth (LB) medium.

### Construction of recombinant plasmid and *S. typhimurium* expressing SspH2-EscI fusion protein

The genomic DNA of bacteria C50041 and O:157 were extracted using the high pure PCR template preparation kit (Takara, Dalian, China) according to the manufacturer’s instructions. The nucleotide sequences of primers for polymerase chain reaction (PCR) were shown in Table 1, with the underlined segments indicating the restriction sites. The 5’-terminal sequence (1–453 bp) of the *sspH2* gene was amplified from the C50041 strain using the primers SspH2-F1 (forward primer) and SspH2-R1 (reverse primer). The 3’-terminal sequence (205–426 bp) of the *escI* gene was amplified from O:157 strain using the primers EscI-F1 (forward primer) and EscI-R1 (reverse primer). The above two purified PCR products were then mixed for the overlap PCR splicing using the primers SspH2-F1 and EscI-R1. All PCR products were subsequently identified via agarose gel electrophoresis. The purified PCR product *sspH2-escI* (729 bp) was cloned into the plasmid pMD20 T and the recombinant plasmid was then transformed into *E.coli* DH5α for amplification. The recombinant plasmid was verified by restriction digestion and DNA sequencing. After digestion with *Nco* I and *Sal* I (Takara), the *sspH2-escI* gene was cloned into the plasmid pYA3334. The recombinant plasmid was named as pYA3334-SspH2-EscI and transformed into *E.coli* X6212. The recombinant plasmid pYA3334-SspH2-EscI was verified by enzyme digestion and DNA sequencing. The plasmid pYA3334-SspH2-EscI was then transformed into *S. typhimurium* X3730 for methylation modification. Finally, the modified plasmid pYA3334-SspH2-EscI was transformed into *S. typhimurium* X4550. The recombinant bacteria were designated as X4550(pYA3334-SspH2-EscI).

**Table 1:**
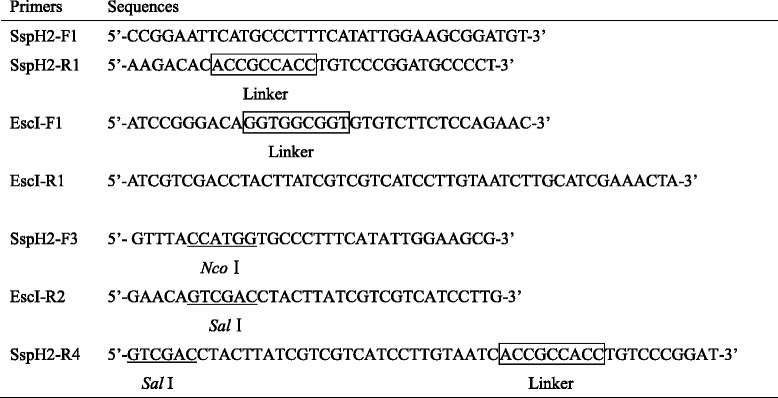
The primer sequences used in this study

The purified PCR product *sspH2* amplified from the C50041 strain using the primers SspH2-F1 (forward primer) and SspH2-R4 (reverse primer) was cloned into the plasmid pYA3334. The recombinant plasmid was named as pYA3334-SspH2 and the corresponding recombinant bacteria was named as X4550(pYA3334-SspH2).

The plasmid pYA3334 was used as a negative control and the corresponding recombinant bacteria was named as X4550(pYA3334).

### Growth curve of recombinant *S. typhimurium* strains

The growth characteristic of recombinant bacteria was performed as previously described [23]. Briefly, single colony of recombinant bacteria was inoculated in LB medium. After being cultured with shaking at 37 °C overnight, 50 μl of bacteria was inoculated in 5 ml LB medium and cultured with shaking at 37 °C. OD600 was measured at different time to obtain the growth curve.

### In vitro infection of mouse peritoneal macrophages

Peritoneal cells were collected by lavaging the mouse peritoneal cavity using RPMI 1640 culture medium supplemented with 10% fetal bovine serum (FBS) (Gibco, Carlsbad, CA). After washing with phosphate buffer saline (PBS), cells were suspended in RPMI 1640 complete medium (RPMI 1640 containing 10% FBS), seeded on 96-well plates, and cultured at 37 °C in 5% CO_2_ for 3 h.

Non-adherent cells were removed and cell density was adjusted to 20,000 cells per well. The adherent cells were pre-stimulated with 1 μg/ml *E. coli* lipopolysaccharide (LPS) (Sigma-Aldrich) to induce the expression of pro-IL-1β. The freshly cultured X4550(pYA3334-SspH2-EscI), X4550(pYA3334-SspH2) and X4550(pYA3334) were centrifuged at 1500 × *g* for 10 min and washed with PBS. The bacteria were resuspended in RPMI 1640 complete medium and added to the LPS-stimulated cells to the desired multiplicity of infection (MOI = 10, 50 and 100, respectively). The cell plate was centrifuged at 500 × *g* for 10 min to enhance the contact of bacteria with the cells. Infected cells were incubated at 37 °C for 30 min. The supernatants were then removed and washed with RPMI 1640 complete medium. Subsequently, RPMI 1640 complete medium containing 100 U/ml penicillin, 100 μg/ml streptomycin, and 1 μg/ml LPS were added to the cells (100 μl/well) to kill the extracellular bacteria. Cells were remained in culture at 37 °C and 5% CO_2_ for four hours [24, 25]. In all experiments, uninfected cells were used as controls. The cell morphology was observed using TS100-F inverted microscope (Nikon, Japan).

After culturing, the supernatants were collected and centrifuged at 2000 × *g* for 5 min to remove all dead bacteria and debris. Quantification of IL-1β and IL-18 was performed using cytometric bead array system (CBA) mouse IL-1β Flex Set and enzyme-linked immunosorbent assay kit (Biosciences, PharMingen, San Diego, CA) according to the manufacturer’s instructions. The flow cytometry was performed using FACSAria flow cytometer with FACSDiva software (Becton-Dickinson Immunocytometry Systems, BDIS, San Jose, CA).

The lactate dehydrogenase (LDH) release was measured using the cytotoxicity detection kit (Roche, Switzerland) according to the manufacturer’s instructions. The relative amount of released LDH was calculated as follows: %released LDH (sample) = (sample − medium background)/(total LDH − medium background) × 100% [26].

Cell plates were washed with PBS and the cells were collected for counting. Then the cells were lysed with lysing solution containing 1 mM phenylmethanesulfonyl fluoride (Westang, Shanghai) according to the manufacturer’s instructions. The intracellular bacteria were counted by coating on the LB agar plate containing NA (20 μg/ml) for culturing.

The intracellular caspase-1 activation were determined by FLICA^TM^ caspase-1 detection kit (Immunochemistry Technoligies Inc., Bloomington, MN) using flow cytometry according to the manufacturer’s instructions.

### In vivo infection of mice

The freshly cultured bacteria were centrifuged at 1500 × *g* for 10 min and washed with PBS. Six-week-old C57BL/6 mice were intravenously injected with X4550(pYA3334), X4550(pYA3334-SspH2) and X4550(pYA3334-SspH2-EscI), respectively. Each mouse was infected with 1× 10^6^ cfu using 100 μl PBS as vehicle. The mice intravenously injected with equivalent PBS were used as controls.

At different time points post-infection, the spleen and liver of mice were harvested to determine bacterial colonization. Briefly, the weight and size of the tissues were recorded. After grinding in 5 ml PBS, the suspension of spleen and liver tissues were ten-fold diluted in PBS and 100 μl suspension were then evenly plated on the LB agar containing nalidixic acid (NA, 20 μg/ml) for CFU enumeration.

Three weeks after infection, the pathological section of spleen and liver of mice were examined using hematoxylin-eosin (HE) staining.

Quantification of IL-6 and TNF-α were performed using CBA mouse inflammation kit (Biosciences, PharMingen, San Diego, CA) according to the manufacturer’s instructions.

### Statistical analysis

Within each experiment, three to four replicate assays were conducted for each treatment and the average value was calculated for final statistical comparisons. All statistical analyses were performed by *t*-tests using SPSS software (Version 13.0 for Windows, Chicago, IL). A value of *P* ≤ 0.05 was considered to be statistically significant.

## Results

### Construction of recombinant plasmid and growth curve of recombinant bacteria

**Fig. 1:**
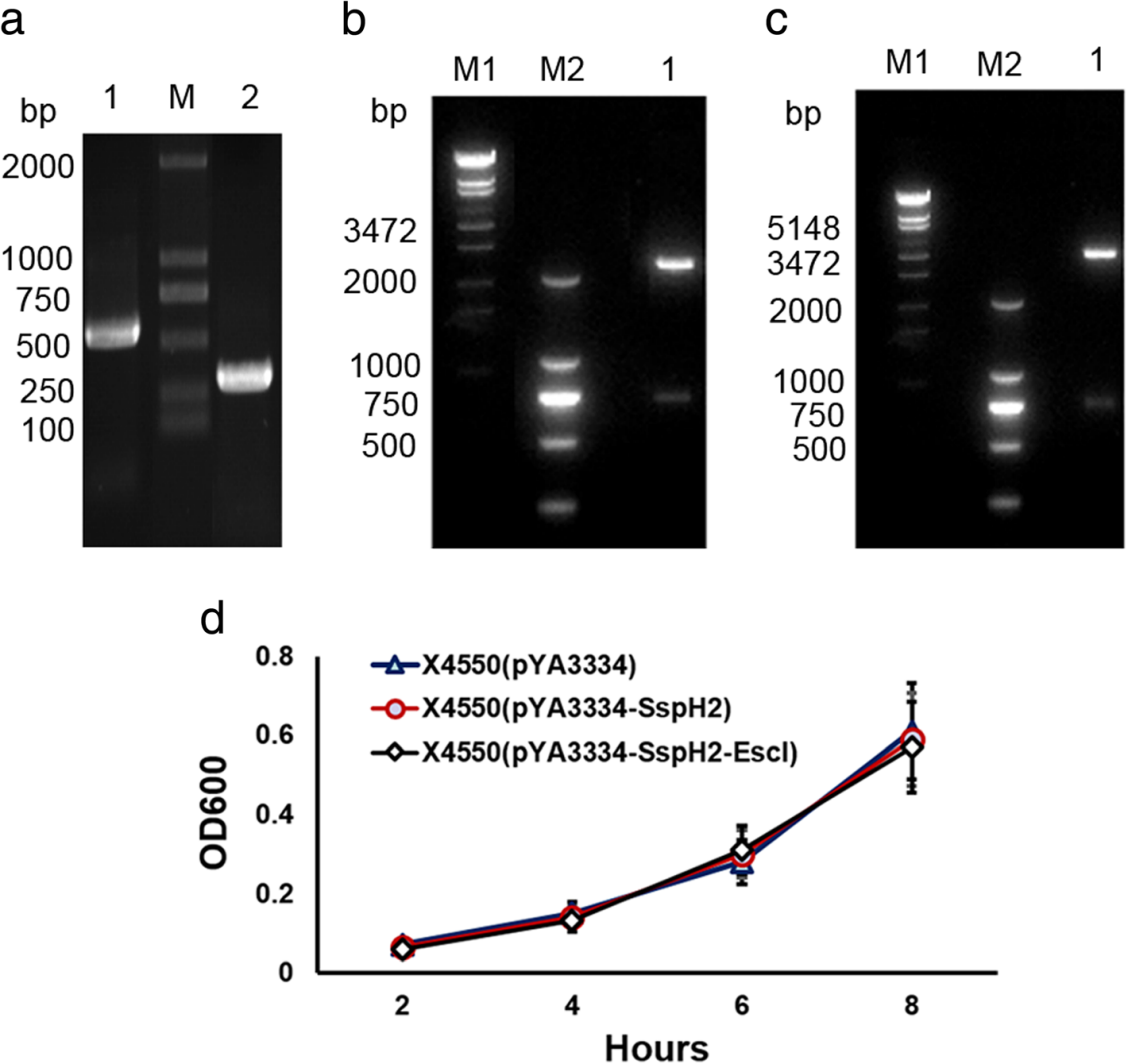
Construction of recombinant bacteria. **a** PCR products. M, Marker; 1, 5’-terminal of *sspH2*; 2, 3’-terminal of *escI*. **b** Recombinant plasmid pMD20 T-SspH2-EscI digested with *Nco* I and *Sal* I. M1-M2, Marker; 1, pMD20 T-SspH2-EscI. **c** Recombinant plasmid pYA3334-SspH2-EscI digested with *Nco* I and *Sal* I. M1-M2, Marker; 1, pYA3334-SspH2-EscI. **d** Growing curve of recombinant bacteria

The PCR products of 5’-terminus of *sspH2* gene (478 bp) and the 3’-terminus of *escI* gene (277 bp) were identified (Fig. 1a). The overlapping PCR product *sspH2-escI* gene was 729 bp as expected. Transformants could grow on LB agar plate without diaminopimelic acid (DAP) (Sigma). The recombinant plasmids subjected to restriction digestion with *Sal* I and *Noc* I were shown (Fig.1b, c). The sequencing results demonstrated that the recombinant plasmid pYA3334-SspH2 and pYA3334-SspH2-EscI was constructed successfully.

The growth states of recombinant bacteria X4550(pYA3334-SspH2-EscI), X4550(pYA3334-SspH2) and X4550(pYA3334) were similar, suggesting that metabolism of the bacteria was not affected by transforming recombinant plasmid into *S. typhimurium* strain (Fig. 1d).

### Pyroptotic cell death of mouse peritoneal macrophages after in vitro infection

**Fig. 2:**
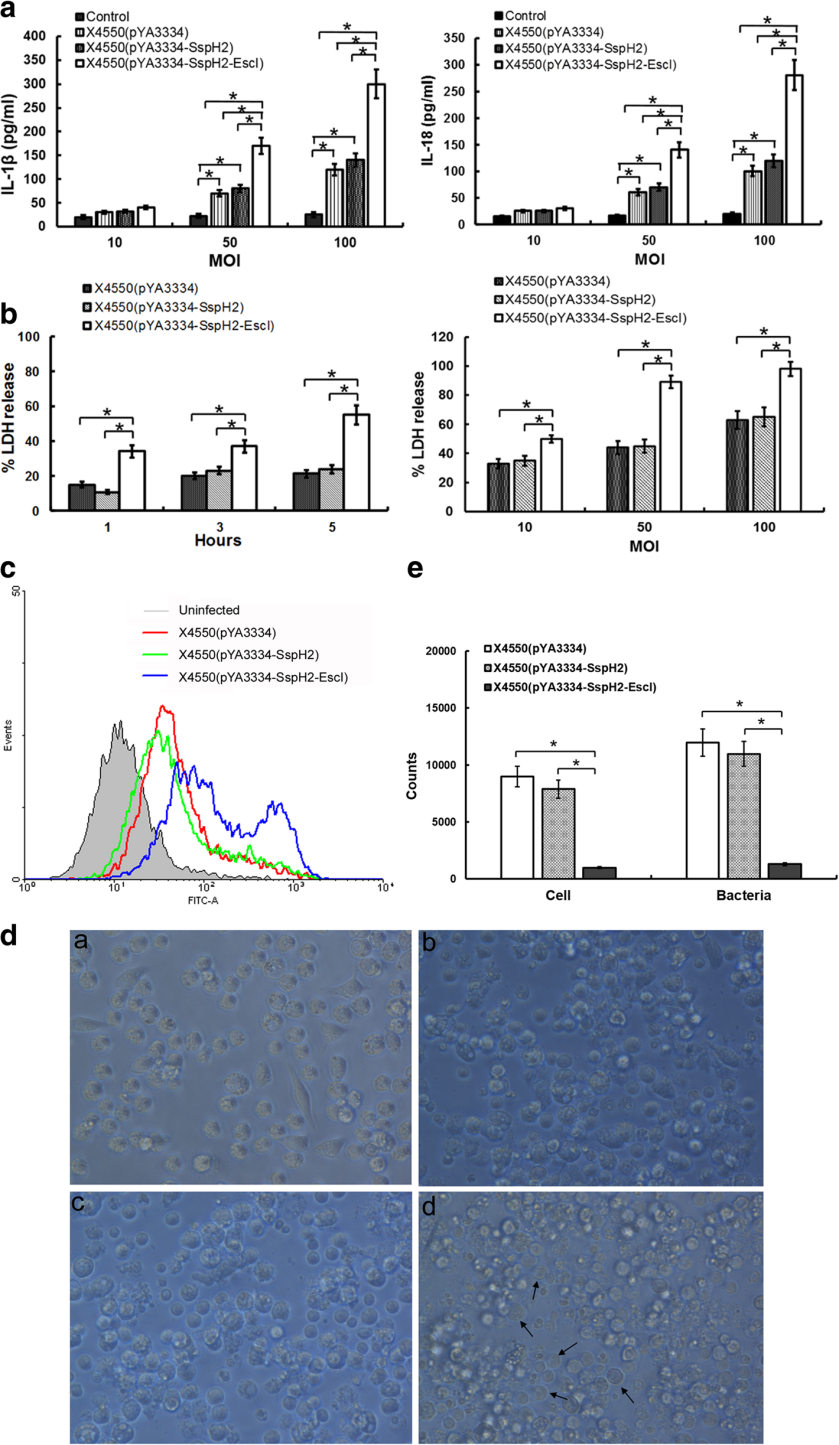
In vitro infection of mouse peritoneal macrophages. C57BL/6 mouse peritoneal macrophages seeded on 96-well plates were pre-stimulated with 1 μg/ml *E. coli* lipopolysaccharide to induce the expression of pro-IL-1β for 3 h. The freshly cultured X4550(pYA3334-SspH2-EscI), X4550(pYA3334-SspH2) and X4550(pYA3334) were then added with the desired multiplicity of infection (MOI). The cell plate was centrifuged to enhance the contact of bacteria with the cells and the infected cells were then incubated for 30 min. The supernatants containing uninfected bacteria were replaced with RPMI 1640 complete medium (100 μl/well) containing 100 U/ml penicillin, 100 μg/ml streptomycin, and 1 μg/ml LPS prior to the start of the subsequent incubation. The uninfected cells were used as control. **a** Supernatant IL-1β and IL-18 levels at 4 h post-infection (hpi) with MOI 10, 50 and 100; **b** LDH release at 1, 3, 5 hpi with MOI 100 and 24 hpi with MOI 10, 50 and 100; **c** Intracellular caspase-1 activation at 1 h hpi with MOI 100; **d** Cell morphology at 24 hpi with MOI 100 (a, uninfection; b, infected with X4550(pYA3334); c, infected with X4550(pYA3334-SspH2); d, infected with X4550(pYA3334-SspH2-EscI), arrows show pyroptotic cell death); **e** The count of cells and intracellular bacteria at 24 hpi with MOI 100. The data shown are representative of three replicate experiments

IL-1β and IL-18 content in the supernatant of peritoneal macrophages following 4 h of infection (MOI = 50 or 100) was significantly higher than those in the unfected control (*P* < 0.05). Notably, infection with X4550(pYA3334-SspH2-EscI) induced significantly more IL-1β and IL-18 secretion from peritoneal macrophages than that induced by X4550(pYA3334-SspH2) or X4550(pYA3334) (*P* < 0.05, Fig. 2a).

LDH release assay indicated that X4550(pYA3334-SspH2-EscI) induced higher cytotoxicity than X4550(pYA3334-SspH2) and X4550(pYA3334) at 1, 3, 5 h post infection with MOI 100 and 24 h post infection with MOI 10, 50 and 100 (*P* < 0.05, Fig. 2b).

X4550(pYA3334-SspH2-EscI) induced higher level of caspase-1 activation than X4550(pYA3334-SspH2) and X4550(pYA3334) after infection. The result at 1 h post infection with MOI 100 was shown in Fig. 2c.

After 24 h, the morphology of X4550(pYA3334-SspH2-EscI)-infected cells (MOI = 100) was found to be markedly poor, and the integrity of the cell membrane was completely lost. Furthermore, the degree of injury induced by X4550(pYA3334-SspH2-EscI) was higher than that indeced by X4550(pYA3334-SspH2) and X4550(pYA3334) (Fig. 2d). Intracellular bacteria in the cells infected with X4550(pYA3334-SspH2-EscI) was significantly lower than that infected with X4550(pYA3334-SspH2) and X4550(pYA3334) (*P* < 0.05, Fig. 2e).

No significant difference was found between the cells infected with X4550(pYA3334-SspH2) and X4550(pYA3334) with regard to the IL-1β and IL-18 secretion, LDH release and intracellular bacterial counts.

### Colonization of recombinant bacteria and pathology in mice

**Fig. 3:**
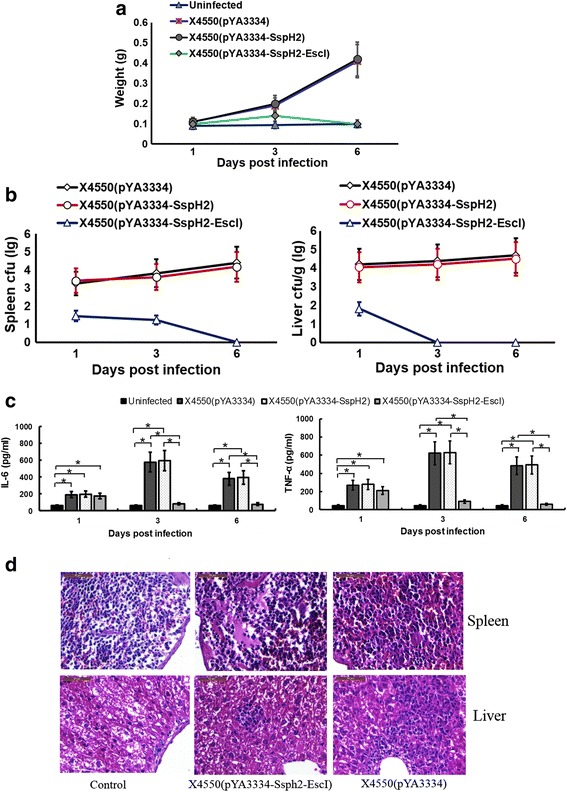
In vivo infection of mice. Six-week-old C57BL/6 mice were intravenously injected with either freshly collected X4550(pYA3334), X4550(pYA3334-SspH2) or X4550(pYA3334-SspH2-EscI), 1× 10^6^ cfu/mouse. Several days later, the weight of spleen (**a**), the bacterial colonization (**b**) in spleen and liver, and the contents of IL-6 and TNF in serum (**c**) were counted. Three weeks post-infection, the spleen and liver (**d**) of the mice were stained with hematoxylin-eosin for pathological assay, all scale bars represent 50 μm. Five mice were used in each treatment. The data shown are representative of three replicate experiments

The spleen and liver of mice infected with X4550(pYA3334) or X4550(pYA3334-SspH2) had significant swelling with the infection time prolongation, when compared with those infected with X4550(pYA3334-SspH2-EscI). Six days after infection, the spleen size and weight of mice infected with X4550(pYA3334) or X4550(pYA3334-SspH2) were approximately four-fold greater than those infected with of X4550(pYA3334-SspH2-EscI). No significant differences were found between the mice infected with X4550(pYA3334-SspH2-EscI) and the uninfected controls (Fig. 3a).

One day after intravenous injection, the bacterial counts of X4550(pYA3334-SspH2-EscI) colonized in mice spleen and liver were significantly lower than those of X4550(pYA3334) and X4550(pYA3334-SspH2) (*P* < 0.05). As infection time extended, the bacteria counts in the spleen and liver of X4550(pYA3334-SspH2-EscI)-infected mice decreased significantly, with no bacteria being detected six days after infection. However, X4550(pYA3334)- and X4550(pYA3334-SspH2)- infected mice were observed to have a significant increase in bacterial counts over the time (Fig. 3b). No significant difference was found between X4550(pYA3334-SspH2) and X4550(pYA3334) with regards to the bacterial counts in the spleen and liver.

All infected mice could secrete IL-6 and TNF-α at 1 day post infection, while the uninfected mice secrete minimal level of IL-6 and TNF-α. Three days after infection, the IL-6 and TNF-α levels in serum of mice infected by X4550(pYA3334-SspH2-EscI) decreased, while those in mice infected with X4550(pYA3334-SspH2) and X4550(pYA3334) significantly increased (*P* < 0.05). No significant difference was found between X4550(pYA3334-SspH2) and X4550(pYA3334) (Fig. 3c).

Three weeks after infection, pathological analysis (Fig. 3d) showed stronger inflammatory responses in the spleen and liver of X4550(pYA3334)- and X4550(pYA3334-SspH2)- infected mice than those of X4550(pYA3334-SspH2-EscI)-infected mice and uninfected controls. Only a few small necrotic foci were found in the liver of X4550(pYA3334-SspH2-EscI)-infected mice, when compared with uninfected controls. In contrast, in the X4550(pYA3334)- and X4550(pYA3334-SspH2)- infected mice, large number of lymphocytes were observed in the splenic sinus, and many necrotic foci containing lymphocytes and necrotic hepatocytes were found in the liver. No significant pathological differences were found between X4550(pYA3334-SspH2) and X4550(pYA3334)-infected mice.

## Discussion

Activation of NLR by microbial components can result in the subsequent activation of inflammasome in macrophages [1, 2] and is beneficial for the defense against intracellular bacteria [7, 8, 27, 28]. This is particularly important for the protection of intestinal mucosa and defense against systemic infection [6, 29]. Currently, the inflammasome mechanism has been predominantly stimulated with peptides in vitro [15]. However, due to the complex regulation by bacteria in the host cells, the responses of host cells against these peptides may be different from their response against the whole bacterium. Thus, it is more practical to study the inflammasome responses through bacterial infection, rather than peptide treatment. It has been reported that the recombinant *Listeria monocytogenes* that can enhance inflammasome response is attenuated and has a protective effect against virulent bacteria challenge [17]. Based on these previous findings, it has been suggested that an attenuated *Salmonella* vaccine candidate that enhances the inflammasome responses can also elevate cellular immunity against subsequent *Salmonella* infection [18]. To test this possibility, we sought to construct a recombinant *Salmonella* strain that can enhance inflammasome activation.

*Salmonella* pathogenicity islands (SPI)-1 and −2 express T3SS1 and T3SS2, respectively [30]. SPI-1 is mainly expressed in the intestines to promote the invasion of *Salmonella* into epithelial cells, while SPI-2 is mainly expressed in host cells to augment the survival of *Salmonella* in macrophages [14]. Reports have shown that NLRC4 can sense *Salmonella* proteins PrgJ and flagellin, which both contain a common C-terminal amino acid sequence [15, 26, 31]. However, over the course of its evolution, *Salmonella* has developed many evasion strategies to prevent NLRC4 detection in macrophages. For instance, SPI-1 and SPI-2 encode the rod proteins PrgJ and SsaI respectively, which form the needle in T3SS basal body [32]. NLRC4 can sense PrgJ, but not SsaI, due to one amino acid difference (V95) in the C-terminus between them [15]. Moreover, flagellin is repressed in the intracellular environment while SPI-2 T3SS is active [16, 33]. Taken together, it has been hypothesized that recombinant *Salmonella* expressing flagellin or PrgJ from a SPI-2 co-regulated promotor can be persistently detected via NLRC4 and completely cleared in vivo [16]. As reported, *Salmonella* effector SspH2 can be translocated by T3SS2 and colocalize with the polymerizing actin cytoskeleton [20]. The recombinant *Salmonella* expressing fusion protein of SspH2 and exogenous antigen can translocate the latter into the cytoplasm of macrophages [20, 21, 34, 35]. Furthermore, the SspH2 N-terminal amino acid sequence is conserved among different *Salmonella* strains and can be used as an efficient delivery vector [20]. EscI protein, the inner rod protein of enteropathogenic *E. coli*, is secreted in the early stage of infection [36, 37] and its C-terminal sequence can activate the NLRC4 inflammasome [15]. In this experiment, the N-terminus of SspH2 and the C-terminus of EscI were selected to construct the recombinant *Salmonella* expressing fusion protein SspH2-EscI.

*Salmonella* lacking *asd* gene has an obligatory requirement for DAP because the *asd* mutant will undergo lysis in environments deprived of DAP. The *asd* + plasmid containing the wild-type *asd* gene can complement the mutants to become a stable balanced-lethal system and be used to express exogenous antigens [38]. The △*crp* △*cya Salmonella* strain X4550 is avirulent and immunogenic in mice, and introduction of *asd* + plasmid pYA3334 into X4550 could completely restore avirulent [22]. It is reported that X4550 still can survive in mice for a long time [23]. Therefore, X4550 is usually used to express exogenous antigen to promote immunity without any antibiotic selection. In this experiment, X4550 was selected as the vector to express and transport fusion protein SspH2-EscI.

The intracellular caspase-1 activation and secretion of IL-1β and IL-18 are essential for inflammasome response in macrophages [7]. The recombinant bacteria expressing SspH2-EscI could significantly promote the secretion of IL-1β and IL-18 and the pyroptotic cell death of macrophages in in vitro infection when compared with bacteria expressing SspH2 only, suggesting that the intracellular recombinant *Salmonella* can successfully express fusion protein SspH2-EscI and the SspH2 N-terminus can be used as a signal to deliver EscI C-terminus into the host cells, resulting in activation of the NLRC4 inflammasome. Furthermore, in in vivo infection, the expression of SspH2-EscI, but not SspH2 alone, could inhibit the colonization of recombinant bacteria, suggesting that reduction of bacterial colonization in mice may be due to the activation of NLRC4 inflammasome by EscI in the cytoplasm.

So far, there are different interpretations about how the inflammasome pathway is used during immune defense [39]. Pyroptosis can lyse the host cells and the pathogen can then be phagocytosed by neutrophils, resulting in bacterial death. As reported, a *sif*A gene-mutated *Salmonella* can destroy the *Salmonella*-containing vacuole due to caspase-11 activation, but not due to the secretion of IL-1β and IL-18. It is also reported that the clearance of *Burkholderia* occurs, in part, due to the secretion of IL-1β and IL-18 after nasal infection. This indicates that pyroptosis is a defense mechanism to clear intracellular bacteria [40]. The anti-infection defense of recombinant *L. monocytogenes* that enhanced the inflammasome activation is due to caspase-1-induced pyroptosis, but not due to the secretion of IL-1β and IL-18 [17]. In this experiment, the inhibition of *Salmonella* colonization in mice several days after intravenous infection may be due to the pyroptosis observed in the earlier stages of infection. The definite mechanism should be further studied in the future using NLRC4¯ mice.

The inflammasome pathway was first named and characterized in 2002 [41] and has since seen great effort to elucidate its mechanism of action. This work can also lead to some applications, especially in the attenuated vaccine design. Because activated inflammasome is not specific to a particular bacteria, this will provide a general platform for the development of vaccine of not only *Salmonella*, but also other intracellular pathogens.

## Conclusions

Through construction of recombinant *Salmonella*, we found that the expression of SspH2-EscI could enhance the activation of inflammasome responses in macrophages and decrease the colonization of bacteria in mice. We speculate that the fusion protein SspH2-EscI may be transported into the cytoplasm of macrophages and then activate NLRC4 inflammasome, which limits the colonization of *Salmonella*.

## Abbreviations

CBA: Cytometric bead array system
FBS: Fetal bovine serum
HE: Hematoxylin-eosin
LB: Luria broth
LDH: Lactate dehydrogenase
LPS: Lipopolysaccharide
NA: Nalidixic acid
NLR: Nucleotide binding domain leucine-rich repeat-containing receptor
NLRC4: NLR family, CARD domain containing-4
PBS: Phosphate buffer saline
PCR: Polymerase chain reaction
SPI: *Salmonella* pathogenicity islands
T3SS: Type III secretion system
